# Supplementary material for: Multimodal imaging and functional analysis of the chick NMDA retinal damage model
Source: PLoS One. 2021 Sep 7;16(9):e0257148. doi: 10.1371/journal.pone.0257148 (PMC8423281; doi:10.1371/journal.pone.0257148)
Supplement: S1 File — (ZIP) [file pone.0257148.s004.zip › SD-OCT/C200727/012/D1-PWk1/C200727-012_OS_R_12_0_REGAVG0000005/Manual_PreDefineThreeByFourGridRadialRetina_1.pdf]

Three By Four Grid Radial Retina Template Report

Patient ID	C200727	Name	See InVivoVue
Exam Date	See InVivoVue	Exam Time	See InVivoVue
Eye	OS	Averaged	N/A
Depth (mm)	1.65	Depth Samples	1024
Pitch (um/pix)	12.000 x 2000.000 x 1.608	Refractive Index	See InVivoVue
Scan Type	Radial 12.000mm x 12.000mm x 1.647mm ,<br>1000 x 6 x 1024		

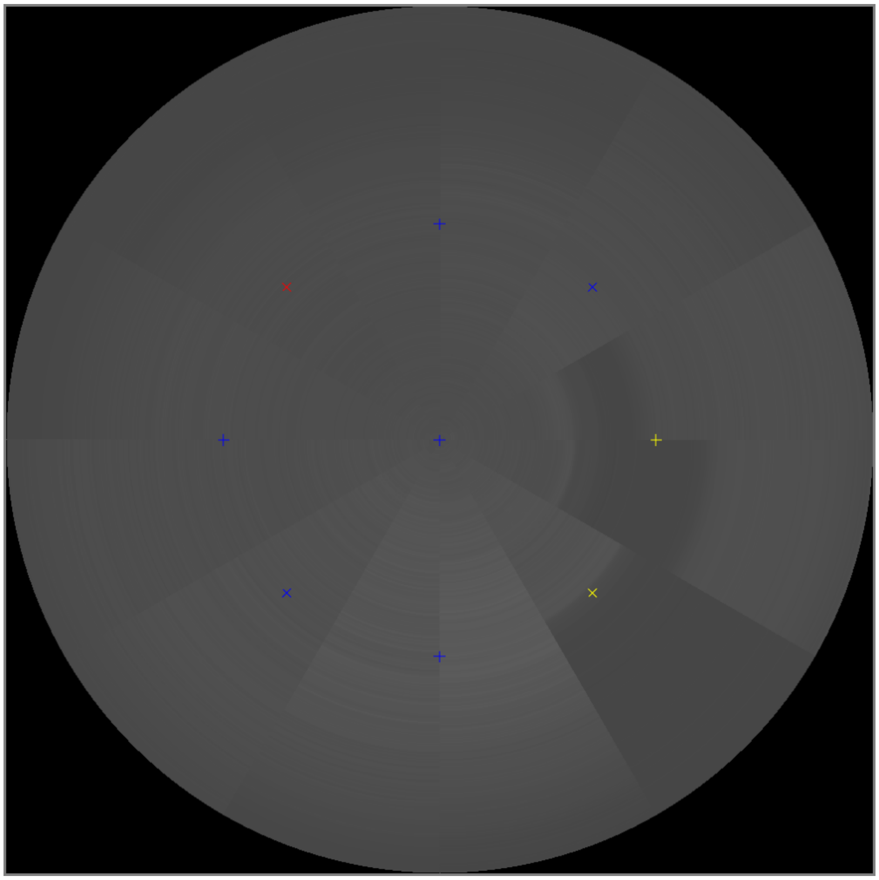

Thickness Metrics

Analysis	Average(mm)	Minimum(mm)	Maximum(mm)	STDEV(mm)
IRNFL - RPE	0.2857	0.2765	0.3058	0.0102
IRNFL - ORNFL	0.0608	0.0325	0.0880	0.0206
ORNFL - OPL	0.1553	0.1342	0.1797	0.0159
OPL - ETPRS	0.0540	0.0487	0.0605	0.0046
